# Cooperation Techniques between LTE in Unlicensed Spectrum and Wi-Fi towards Fair Spectral Efficiency

**DOI:** 10.3390/s17091994

**Published:** 2017-08-31

**Authors:** Vasilis Maglogiannis, Dries Naudts, Adnan Shahid, Spilios Giannoulis, Eric Laermans, Ingrid Moerman

**Affiliations:** IDLab, Department of Information Technology, imec, Ghent University, Technologiepark Zwijnaarde 15, B-9052 Ghent, Belgium; dries.naudts@ugent.be (D.N.); adnan.shahid@ugent.be (A.S.); spilios.giannoulis@ugent.be (S.G.); eric.laermans@ugent.be (E.L.); ingrid.moerman@ugent.be (I.M.)

**Keywords:** LTE, Wi-Fi, cooperation, coexistence, cognitive radio, spectral efficiency, fairness

## Abstract

On the road towards 5G, a proliferation of Heterogeneous Networks (HetNets) is expected. Sensor networks are of great importance in this new wireless era, as they allow interaction with the environment. Additionally, the establishment of the Internet of Things (IoT) has incredibly increased the number of interconnected devices and consequently the already massive wirelessly transmitted traffic. The exponential growth of wireless traffic is pushing the wireless community to investigate solutions that maximally exploit the available spectrum. Recently, 3rd Generation Partnership Project (3GPP) announced standards that permit the operation of Long Term Evolution (LTE) in the unlicensed spectrum in addition to the exclusive use of the licensed spectrum owned by a mobile operator. Alternatively, leading wireless technology developers examine standalone LTE operation in the unlicensed spectrum without any involvement of a mobile operator. In this article, we present a classification of different techniques that can be applied on co-located LTE and Wi-Fi networks. Up to today, Wi-Fi is the most widely-used wireless technology in the unlicensed spectrum. A review of the current state of the art further reveals the lack of cooperation schemes among co-located networks that can lead to more optimal usage of the available spectrum. This article fills this gap in the literature by conceptually describing different classes of cooperation between LTE and Wi-Fi. For each class, we provide a detailed presentation of possible cooperation techniques that can provide spectral efficiency in a fair manner.

## 1. Introduction

Over the past few years, the technological growth combined with the proliferation of wireless devices such as sensors, smartphones, laptops and wearable technology has changed the way that information is exchanged. The number of interconnected devices and the number of Heterogeneous Networks (HetNets) increase rapidly. The development and the consolidation of wireless sensor networks has further contributed to the increase of the wireless traffic, as often they consist of hundreds to thousands of wireless sensor nodes. The first-generation Internet has evolved into the Internet of Everything, where massive amounts of information are exchanged between devices using different types of mainstream and well-established wireless technologies such as LTE, Wi-Fi, IEEE 802.15.4 and Bluetooth. Recently, the sub-gigahertz bands have been extensively exploited by wireless technologies that offer wide ranging communications, such as LORA, SIGFOX and 802.11ah. Moreover, high frequency bands such as mmWave are also being used for multi-gigabit speeds (IEEE 802.11ad). According to Qualcomm, the amount of wireless traffic is expected to further increase by a factor of 1000 by 2020 [1]. Additionally, Cisco’s latest forecast expects that the traffic from wireless and mobile devices will exceed the overall wired traffic by 2019 [2]. Based on these predictions, the wireless network capacity will soon become a bottleneck for the massive growth of wireless traffic.

The 5G community has already started investigating solutions that can lead to the 1000× challenge. These solutions include among others, enhanced massive Multiple-Input Multiple-Output (MIMO), carrier aggregation, higher-order modulation schemes such as 64-Quadrature Amplitude Modulation (QAM) or 256-QAM, cloud computing services and advanced network architecture modifications. At the same time, the adoption of LTE from different applications gains ground, as it is a technology that approaches the Shannon limit and can contribute significantly to solving the network capacity challenge.

Recently, key players of the mobile world have proposed standards to the 3rd Generation Partnership Project (3GPP), which allow LTE operation in the unlicensed spectrum. To this end, 3GPP announced the operation of LTE Licensed-Assisted Access (LTE LAA) [3], as an enhancement within 3GPP LTE Release 13. LTE LAA will allow operators to use a secondary cell operating in the unlicensed spectrum, alongside the primary cell operating in the licensed band they own. The carrier aggregation that has been introduced in 3GPP LTE Release 10 [4] will be used to enable this feature. There are two predominant proposals for LTE LAA. According to the first one, the secondary cell will operate in the unlicensed spectrum for supplemental Downlink (DL) traffic only, while Uplink (UL) traffic will be transmitted over the operator’s licensed spectrum. In the second proposal, the secondary cell operating in the unlicensed spectrum can be used for both DL and UL LTE traffic.

On the other hand, leading wireless stakeholders other than mobile operators are taking the first steps towards exploitation of LTE in the unlicensed spectrum as a standalone wireless solution complementary to Wi-Fi. To this end, they formed the MulteFire Alliance [5]. Their target is to decouple LTE from the operators, so it can be deployed by Internet service providers (ISPs) enterprises, building owners, cable companies, etc.

**Figure 1:**
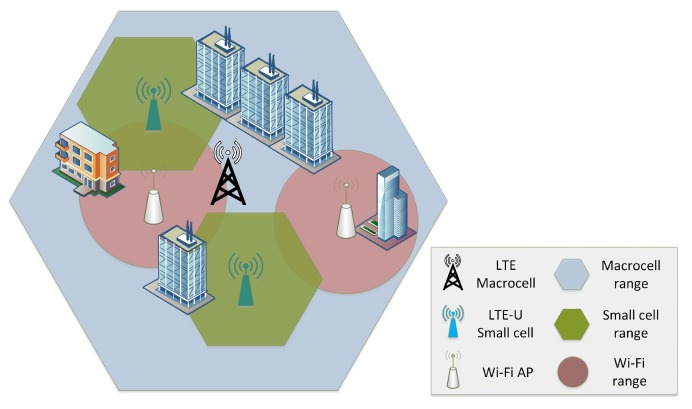
Deployment of LTE in the unlicensed spectrum next to the current infrastructure.

Figure 1 indicates how LTE in the unlicensed spectrum could be deployed next to the current wireless infrastructure, where an LTE-U small cell could be either an LTE LAA small cell, controlled by a mobile operator, or a small cell operating solely in the unlicensed spectrum without mobile operator control.

LTE is a technology that has been initially designed to operate in the licensed spectrum. Hence, it assumes that it can exclusively use the whole assigned spectrum, and therefore, it does not incorporate any techniques for harmonious coexistence with other possible co-located technologies. It is clear that introducing LTE in the unlicensed spectrum as is will cause significant coexistence issues with other well-established technologies such as Wi-Fi, IEEE 802.15.4 or Bluetooth. This means that LTE will have a negative impact on the performance of traditional unlicensed technologies in terms of throughput, latency and other Quality of Service (QoS) guarantees [6], affecting their applications such as wireless (sensor) networks, Device-to-Device (D2D) and Machine-to-Machine (M2M) communications. To this end, research has focused on the design and evaluation of coexistence techniques for LTE, in order to enable fair spectrum sharing with other technologies operating in the unlicensed spectrum, and in particular with Wi-Fi. On the other hand, much less attention has been paid to cooperation techniques between the two technologies. The networks that participate in a cooperation scheme are able to exchange information directly or indirectly (via a third-party entity) in order to improve the efficiency of spectrum usage in a fair way.

In this article, we distinguish two different classes of cooperation between LTE and Wi-Fi, and for each class, we propose and analyze potential cooperation techniques that can be applied. For each cooperation technique, we analyze the advantages and disadvantages regarding the design and deployment complexity, the flexibility and the efficiency they could offer. The proposed techniques can contribute to the open discussion regarding the standardization process of the LTE operation in the unlicensed spectrum. The main contribution of this work is summarized as follows:

- Classification of techniques that can be applied on co-located LTE and Wi-Fi networks
- Detailed analysis of the current state of the art regarding LTE in the unlicensed spectrum and Wi-Fi covering:
  ‒ Analysis of the standard LTE and Wi-Fi protocols
  ‒ The regional regulations for the unlicensed spectrum
  ‒ The impact of LTE on Wi-Fi without applying any coexistence technique
  ‒ The current approaches for coexistence between LTE and Wi-Fi
- Analysis of the different concepts of cooperation between LTE and Wi-Fi and potential techniques that can be applied for realizing each concept
- Comparison and feasibility of the different presented concepts

The remainder of the article is organized as follows. Section 2 presents a classification of the techniques that can be applied when LTE and Wi-Fi networks are co-located and operate in the same (unlicensed) frequency band. Section 3 discusses the current state of the art for LTE operation in the unlicensed spectrum. Next, in Section 4, we analytically present the concept of direct cooperation between LTE and Wi-Fi via in-band energy level patterns and showcase possible cooperation techniques. Section 5 presents the concept of cooperation between LTE and Wi-Fi using indirect communication through a third-party entity and describes possible cooperation techniques. In Section 6, we compare the proposed concepts and techniques. Finally, in Section 7, we conclude the paper and discuss plans for future work.

## 2. Taxonomy of Techniques for Co-Located LTE and Wi-Fi Networks

**Figure 2:**
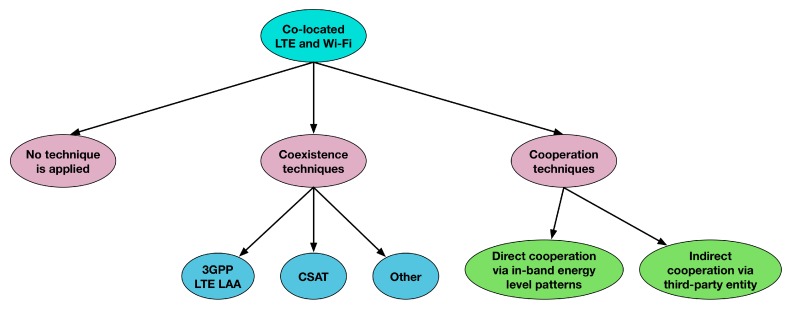
Taxonomy of the techniques that can be applied to co-located LTE and Wi-Fi networks.

This section presents a taxonomy of techniques that can be applied when an LTE network is co-located with a Wi-Fi network and both networks operate in the same frequency band. This taxonomy is presented in Figure 2. As can be seen, co-located LTE and Wi-Fi networks can be classified into three big categories depending on the techniques that are applied between them.

In the first category, the networks operate next to each other in the way they were initially designed, without any technique that improves the symbiosis between them. This is the worst type of co-location scenario as LTE transmissions result in a severe impact on Wi-Fi [7].

The other two categories classify two co-located networks based on whether the applied technique aims to provide coexistence or cooperation between the networks. Coexistence and cooperation are two terms that differ significantly. With the term coexistence, we refer to methodologies that enable peaceful operation of a wireless technology next to another. The technologies must respect each other, as well as the regional regulations and seek equal opportunities to access the wireless medium, under the condition that there is no exchange of any information between different technologies. On the other hand, the cooperation term refers to methodologies that seek collaboration among the technologies towards harmonious coexistence and optimal spectrum usage by exchanging information. The cooperation between different technologies is in line with the 5G vision, where all of the available wireless technologies will act towards enhancing the user experience.

As can be seen from Figure 2, coexistence techniques include the 3GPP LTE LAA mechanism and other techniques that are described later in Section 3. Although coexistence techniques between LTE operating in the unlicensed spectrum and Wi-Fi have been studied widely, the literature, to the best of our knowledge, consists of only a limited number of studies focusing on cooperation techniques among the two technologies. This paper targets covering this gap by classifying the possible cooperation schemes and proposing potential techniques that can be applied in each category. The different cooperation techniques can be classified into the following two big categories, based on the way that the participating networks communicate with each other:

- Direct cooperation via in-band energy level patterns
- Indirect cooperation via a third-party entity

The first category includes techniques that make use of one or multiple in-band energy patterns in order to perform technology identification, inform about their actions or achieve synchronization between multiple networks that participate in the cooperation scheme.

The second category refers to the ability for different wireless technologies to exchange messages via a third-party entity, in order to maintain synchronization and explicitly describe their characteristics and requirements.

## 3. State of the Art

### 3.1. LTE and Wi-Fi in a Nutshell

This section briefly describes the mechanisms that LTE and Wi-Fi use to transmit in the way that they were initially designed. The analysis of the core differences among these mechanisms will give us the insight to understand in depth the reasons why LTE cannot operate next to Wi-Fi without appropriate coexistence and cooperation mechanisms. Moreover, it will be used as a basis for the subsequent description of the different cooperation protocols.

#### 3.1.1. LTE

**Figure 3:**
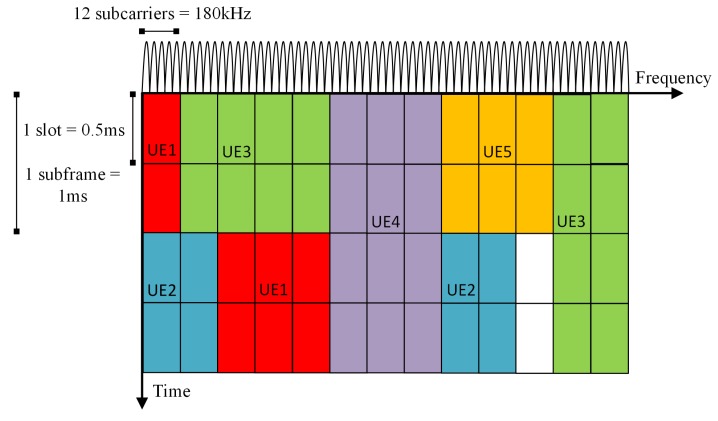
LTE time-frequency structure and user traffic scheduling. Each color represents a different UE that is scheduled by the Base Station.

Upon a transmission, LTE uses the multi-user versions of the Orthogonal Frequency Division Multiplexing (OFDM) digital modulation scheme, called Orthogonal Frequency Division Multiple Access (OFDMA) for the DL and Single-Carrier Frequency Division Multiple Access (SC-FDMA) for the UL [8]. The available spectrum is divided into subcarriers, and each subcarrier occupies 15-kHz of bandwidth. The time domain is organized in timeslots of 0.5 ms in duration. One timeslot corresponds to seven OFDM symbols when the normal Cyclic Prefix (CP) is used and six OFDM symbols when the extended CP is used. Combining the subcarriers and timeslots, LTE defines the Resource Block (RB). The RB is a unit of transmission resource and consists of one slot in the time domain and 12 subcarriers in the frequency domain. An LTE radio frame has a duration of 10 ms and consists of 10 sub-frames, each of which lasts 1 ms corresponding to two slots. Figure 3 shows the structure of resource blocks in the frequency-time domain and how they are scheduled for different User Equipment (UE).

LTE sends user traffic in the DL and in the UL using the Physical Downlink Shared Channel (PDSCH) and the Physical Uplink Shared Channel (PUSCH), respectively. In addition to user traffic, several RB are allocated for control traffic in dedicated channels. Such control traffic includes among others the transmission of synchronization signals and reference signals.

LTE is a scheduled technology designed to operate in the licensed spectrum. Therefore, it does not require sensing the medium before transmission. The scheduling in LTE is performed by the LTE base station named the evolved NodeB (eNB) on a subframe basis. That means that every 1 ms, the assignment of the subframes to the active UEs can change.

#### 3.1.2. Wi-Fi

Wi-Fi uses the Distributed Coordination Function (DCF) as the fundamental mechanism to access the medium and is designed to be asynchronous and decentralized [9]. Additionally, it uses the OFDM digital modulation scheme that divides the spectrum into multiple OFDM subcarriers spanning 20 MHz or multiple spectral portions of 20 MHz. In order to sense and gain access to the medium, Wi-Fi uses the Carrier Sensing Multiple Access with Collision Avoidance (CSMA/CA) mechanism before a packet transmission. According to this contention-based protocol, a Wi-Fi node first has to listen to the shared medium to determine if there are other ongoing transmissions. This procedure is known as Clear Channel Assessment (CCA). The node senses the channel for a DCF Inter-Frame Space (DIFS) duration. Only when the channel is estimated as idle is the node permitted to transmit.

CCA consists of two functions named Carrier Sense (CS) and Energy Detection (ED). The CS function refers to the ability of the receiver to detect and decode a received Wi-Fi preamble. According to the specifications, if the power of the detected signal is higher or equal to −82 dBm for the 20-MHz bandwidth, then CCA reports the channel as busy for the timeslot that is indicated in the frame’s Physical Layer Convergence Protocol (PLCP) length field [9]. This field contains either the time in µs that is required for the Medium Access Control (MAC) Protocol Data Unit (MPDU) payload transmission or the number of octets carried in the frame MPDU payload, which is used to compute the time required for the MPDU transmission.

If the received signal cannot be decoded, the ED function is used. The ED function refers to the ability of the receiver to detect the energy level in the operating channel based on non-Wi-Fi signals that are sensed, introducing interference, or based on corrupted Wi-Fi transmissions that cannot be decoded. If the energy level is higher than −62 dBm for a 20-MHz bandwidth, then CCA reports the channel as busy. ED senses the channel every time slot to estimate the corresponding energy level [9].

**Figure 4:**
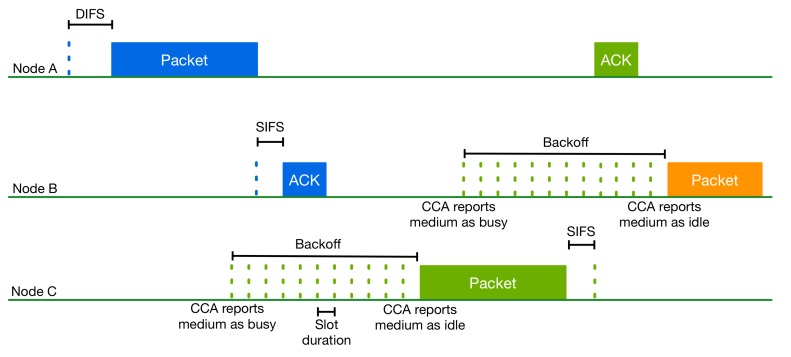
The 802.11 CSMA/CA procedure.

In case the channel is sensed as idle for a DCF Inter-Frame Spacing (DIFS) period, then the Wi-Fi node can transmit. Otherwise and also prior to attempting to transmit again immediately after a successful transmission, the node has to postpone its transmission and wait for a free DIFS plus a random backoff time to avoid packet collisions. After a transmission, the node waits for an acknowledgment during a Short Inter-Frame Space (SIFS) period. If the acknowledgment is not received after this timeout period, the node schedules a retransmission after a new exponential backoff period and until the maximum number of retransmissions is reached. Figure 4 illustrates the CSMA/CA algorithm described above.

### 3.2. Regional Regulations

In order to enable fair coexistence among LTE and Wi-Fi, research has been focusing on designing coexistence techniques that will allow LTE to operate in the unlicensed spectrum, respecting the different regional regulations. Concurrently, these techniques aim to fairly share the medium with other well-established technologies like Wi-Fi. For instance, the European Telecommunications Standards Institute (ETSI) defines requirements [10] that should be fulfilled by each technology that operates in the unlicensed spectrum. These requirements among others include:

- CCA before transmission together with timing requirements for each CCA phase
- Maximum antenna gain
- Transmission power limitations

Table 1 summarizes the regional regulations that must be obeyed by a technology that operates in the unlicensed spectrum. In the table, DFS stands for Dynamic Frequency Selection and TPC stands for Transmit Power Control.

### 3.3. Impact of LTE Operating in the Unlicensed Spectrum on Wi-Fi

The previous section has described the different operational methods for LTE and Wi-Fi. It is clear that introducing LTE into the unlicensed spectrum, in the way it was originally designed, will have a significant impact on the performance (throughput, latency, packet loss, spectral efficiency) of a co-located Wi-Fi network. As LTE can schedule traffic without sensing the medium for ongoing transmissions, it can interfere with Wi-Fi within the overlapping spectrum. Hence, the CCA mechanism of Wi-Fi, and more specifically the ED function, will force Wi-Fi to backoff. This impact can become even higher by consecutive LTE transmissions. Then, LTE will either seriously degrade the signal quality of Wi-Fi due to collisions (if the LTE signal power is below the ED threshold, but still high enough to interfere with the Wi-Fi transmissions), or lead to Wi-Fi starvation, as it will be forced to backoff continuously.

Several studies have evaluated the impact of traditional LTE on Wi-Fi, when both technologies operate in the same frequency band without any coexistence mechanism being applied. In our previous work [7], we studied the impact of LTE operating in the unlicensed spectrum on Wi-Fi using Off The Shelf (OTS) hardware equipment [11]. The experiments were performed on the LTE and Wi-Fi infrastructure of the W-iLab2 testbed at imec [12]. Three different levels of LTE signal have been examined, representing different possible levels of LTE impact on Wi-Fi. The results show that the Wi-Fi performance, in terms of throughput and latency, can be significantly affected by LTE.

Other approaches evaluate the Wi-Fi performance degradation based on simulations and mathematical models. In [13,14], the authors evaluate the performance of both LTE and Wi-Fi when both technologies operate in a shared band. All studies come to the same conclusion, namely that LTE causes a serious impact on Wi-Fi, when both operate in the same band without any coexistence mechanism among them and no medium sensing mechanism enabled at the LTE side.

### 3.4. Proposed Coexistence and Coordination Techniques

#### 3.4.1. LTE LAA Approach

**Figure 5:**
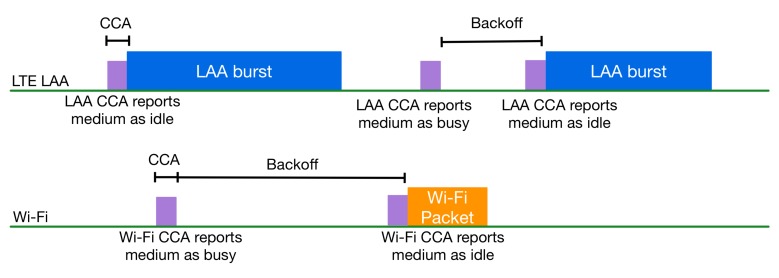
LTE Licensed-Assisted Access (LAA) and Wi-Fi coexistence.

Towards a coexistence technique that respects the regional regulations, 3GPP announced the LTE LAA standards in Release 13, including the description of a Listen Before Talk (LBT) procedure (also known as CCA) [15]. Initially, LTE LAA is scheduled to operate within the 5-GHz unlicensed spectrum and for DL traffic only, but in a later phase, it is expected to be extended to the 2.4-GHz unlicensed band, as well as for both DL and UL traffic. Initially, an eNB will be able to activate and deactivate a secondary cell operating in the unlicensed spectrum. Through this cell, only data traffic (via the PDSCH) can be sent, while the LTE control signals and the UL traffic (PUSCH) will be transmitted via the licensed anchor. The eNB must perform the LBT procedure and sense the channel prior to a transmission in the unlicensed spectrum. When the channel is sensed as busy, the eNB must defer its transmission by performing an exponential backoff. If the channel is sensed to be idle, it performs a transmission burst with a duration from 2–10 ms, depending on the channel access priority class. The authors in [16] analytically describe the LTE LAA procedure. They provide an overview of the LAA mechanism including the motivation and use cases where it can be applied. Additionally, they present a coexistence evaluation methodology and results, which have been contributed by 3GPP. Figure 5 shows the LTE LAA and Wi-Fi coexistence in the same channel in the unlicensed spectrum.

#### 3.4.2. LTE CSAT Approach

**Figure 6:**
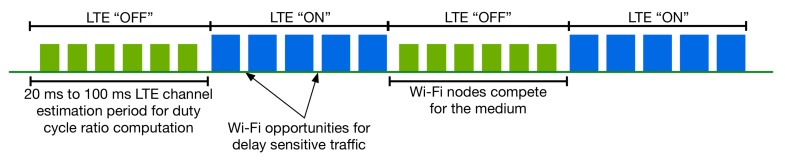
Carrier Sensing Adaptive Transmission (CSAT) duty cycle periods.

In regions such as the U.S., China and South Korea, where an LBT procedure is not required by the local regulations, different types of coexistence techniques can be applied. Carrier Sensing Adaptive Transmission (CSAT) [17], proposed by Qualcomm, is a technique that can enable coexistence among LTE and Wi-Fi based on minor modifications of the 3GPP LTE Release 10/11/12 Carrier Aggregation protocols [4]. CSAT introduces the use of duty cycle periods and divides the time into LTE “ON” and LTE “OFF” slots. During the LTE “OFF” period, also known as the “mute” period, LTE remains silent, giving the opportunity to other coexistent networks, such as Wi-Fi, to transmit. During the LTE “ON” period, LTE accesses the channel without sensing it before a transmission. Moreover, CSAT allows short transmission gaps during the LTE “ON” period to allow for latency sensitive applications, such as VoIP in co-located networks. In CSAT, the eNB senses the medium for a time period ranging from tens of ms up to 100 ms and according to the observed channel utilization (based on the estimated number of Wi-Fi Access Points (APs)) defines the duration of the LTE “ON” and LTE “OFF” periods [17]. Figure 6 depicts the CSAT duty cycle periods.

#### 3.4.3. Other Related Work

Coexistence between LTE and Wi-Fi in the unlicensed spectrum has attracted the attention of the mobile and the research community. There are several proposed mechanisms, trying to achieve fair coexistence between the two technologies. These mechanisms are evaluated on the success of providing the desired fairness.

In [18], the authors discuss a preliminary design of a semi-distributed LTE in the unlicensed spectrum scheme, where the eNB senses the carrier before a transmission in a similar way to Wi-Fi. They use a method that exploits the LTE Almost Blank Subframes (ABS). The ABS were initially designed to enhance Inter-Cell Interference Coordination (eICIC) as part of 3GPP LTE Release 10 [19]. The proposed method evaluates different duty cycles, different distances between an eNB and an AP and different numbers of cells. Different ABS patterns are also studied.

In [20], the authors propose an LTE subframe design consisting of three phases named the data transmission phase, the mute phase and the sensing and reservation phase. Based on this subframe design, they proposed three schemes to enable coexistence among LTE and Wi-Fi. The first scheme assumes a fixed mute duration. The second scheme uses a randomized mute duration. The third scheme introduces a random backoff counter during the mute period. The three proposed schemes are evaluated through simulations. The results show that the scheme that uses a random mute duration offers better overall throughput performance, while the scheme that uses a random backoff counter results in smaller throughput difference between LTE and Wi-Fi.

The authors in [21] describe an analytical framework for interference characterization of Wi-Fi and LTE. Initially, a first model is described for single LTE and single Wi-Fi AP separated by a specific distance. The results show that Wi-Fi performance is significantly decreased compared to LTE for which the degradation is minimal. They observe that the conventional perception of the inverse proportion of throughput to inter-AP distance is not valid for LTE-Wi-Fi co-channel deployment. A second model with many LTE and Wi-Fi systems is also described. The results show that the overall system throughput first increases and then decreases with growing density. Finally, in order to increase the individual Radio Access Technology (RAT) and system throughput, random channel assignment, intra- and inter-RAT channel coordination are considered. In the intra- and inter-RAT channel coordination schemes, the channel is allocated at an AP as a graph multi-coloring problem. The results show 3.5–5× gains in system capacity. In this technique, the networks do not exchange specific information in order to optimize the offered QoS, but different frequencies are assigned to them in order to avoid overlapping frequencies between co-located networks.

In [22,23], the authors use Q-Learning techniques to achieve the desired coexistence. In [22], they propose a Q-Learning-based dynamic duty cycle selection mechanism for configuring LTE transmission gaps. LTE LAA and Wi-Fi performance using a fixed transmission gap is evaluated and then compared with the proposed Q-Learning mechanism. Simulation results show that the proposed scheme enhances the overall capacity performance. The authors in [23] propose a Q-Learning mechanism for advanced learning of the activity within the unlicensed band resulting in efficient coexistence between LTE LAA and Wi-Fi. As a next step, the coexistence is further enhanced through a double Q-Learning method that takes into account both discontinuous transmission and transmit power control of LTE to improve both LTE and Wi-Fi performance.

Coexistence of LTE and Wi-Fi when LTE uses an LBT procedure is studied in [24,25,26,27]. The authors in [24] propose an adaptive LBT protocol for LTE LAA. This protocol enhances the coexistence with Wi-Fi and increases the overall system performance. The protocol consists of two different mechanisms named on-off adaptation for channel occupancy time and short-long adaptation for idle time. The first mechanism is responsible for adapting the channel occupancy time of LTE based on the load of the network, while the second one adapts the idle period based on the Contention Window (CW) duration of Wi-Fi. The authors in [25] propose an LBT mechanism for LTE LAA that aims to share the medium in a fair way towards the increase of the overall system performance. This work includes both a mathematical analysis and a validation via simulation of the proposed LBT scheme. The results show that a proper selection of LAA channel occupancy and the backoff counter can increase the performance of Wi-Fi. In [26], the authors study the coexistence among LTE LAA and Wi-Fi using the LBT category four-channel access scheme. The behavior of LAA eNB is modeled as a Markov chain, and the obtained throughput is adopted as the performance metric. The proposed LBT scheme uses an adaptive CW size for LTE LAA. According to the results, the proposed scheme outperforms the fixed CW size. In [27], the authors examine how LTE cells in the unlicensed spectrum from different operators can adjust their CW in order to tune the LBT algorithm and provide coexistence both with Wi-Fi and among themselves in an altruistic way. The interaction of LTE cells in the unlicensed spectrum is studied using a coalition formation game framework, which is based on the Shapley value.

In [28], the authors present an analytical model for evaluating the performance of coexistence between LTE and Wi-Fi. This model has been used to obtain baseline performance measures. The results of the model have been partially validated via experimental evaluation using Universal Software Radio Peripheral (USRP) platforms. Moreover, the authors propose an inter-network coordination with logically centralized radio resource management across LTE and Wi-Fi as a solution to improve coexistence.

The authors in [29] propose two non-coordinated and two coordinated network management approaches to enable coexistence. Regarding the non-coordinated techniques, the first one proposes eNB to perform LBT on different channels and to switch to a different channel after a transmission, while the second proposes LTE to offer transmission opportunities of variable duration to Wi-Fi after a transmission based on the occupancy of the medium. Concerning the coordinated methodologies, the first one proposes a Network Function Virtualization (NFV) interconnection to combine the Wi-Fi network and the service provider of LTE in the unlicensed spectrum. This way, channel selection and seamless transfer of resources between the two technologies can be enabled, using the in-the-cloud control of distributed APs. The second method proposes the management of coexistence using the X2 interface among the eNBs. The eNBs can exchange information and schedule ABS in different subframes, thus giving more opportunities to any Wi-Fi network that is located potentially within their proximity. In the aforementioned schemes, the different RATs are under the control of the same mobile operator. The coordination between the wireless technologies targets the enhancement of the overall QoS that the operator offers (e.g., perform load balancing via frequency coordination).

Finally, the authors in [30] provide a detailed survey of the coexistence of LTE and Wi-Fi on 5 GHz with the corresponding deployment scenarios. They provide a detailed description of the coexistence-related features of LTE and Wi-Fi, the coexistence challenges, the differences in performance between the two different technologies and co-channel interference. They extensively discuss the proposed coexistence mechanisms between LTE and Wi-Fi in the current literature. Furthermore, the survey discusses the concept of the scenario-oriented coexistence, in which coexistence-related problems are solved according to different deployment scenarios.

Although the coexistence between LTE operating in the unlicensed spectrum and Wi-Fi is being investigated extensively, little attention is given to studies that investigate cooperation scenarios among the two technologies. As has been discussed in Section 2, in this paper, we distinguish two different types of cooperation between LTE in the unlicensed spectrum and Wi-Fi. Furthermore, for each solution, we propose and describe different techniques that can lead to efficient and fair spectrum use.

## 4. Direct Cooperation via In-Band Energy Level Patterns

### 4.1. Introduction

**Figure 7:**
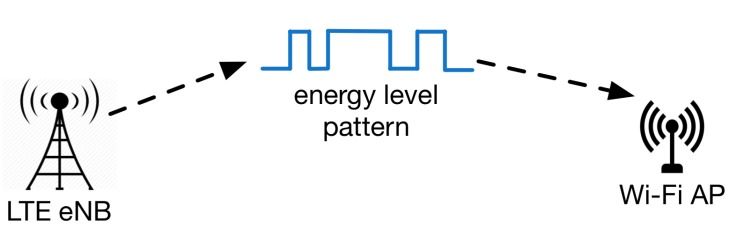
LTE identification via the predefined energy level pattern.

This section describes cooperation techniques between co-located LTE and Wi-Fi networks that operate in the same frequency band, using in-band pattern recognition in order to enhance the spectral efficiency of the coexisting networks. A cooperation scheme that uses in-band pattern recognition can be applied, when the co-located networks do not have the ability to communicate between each other (e.g., via a coordinator) in order explicitly to express their requirements. The in-band pattern recognition methodology allows direct cooperation between different wireless technologies, as it can be used for technology identification and inter-RAT synchronization. Moreover, a wireless technology can use one or more in-band special patterns in order to inform other technologies about different actions that it performs. Upon the recognition of such a pattern, a wireless network will be able to adapt its behavior towards an increased performance (e.g., higher throughput) and more advanced spectrum usage. Figure 7 depicts an example of an in-band pattern recognition. In this example, a predefined energy level pattern is transmitted by the LTE eNB. This pattern is used for the identification of the LTE network by a Wi-Fi AP.

For a technique of such a nature, the complexity of the design and the implementation is relatively low, as only small modifications of the current protocols of each wireless technology are required in order to transmit and interpret such energy level patterns. Nevertheless, the low complexity of the methodologies implies also a limited flexibility, meaning that upon sensing a co-located wireless technology, each network takes some predefined actions that can contribute to more efficient spectrum sharing and/or performs readjustment and tuning of existing coexistence techniques.

### 4.2. Enhanced CSAT

One negative aspect of the CSAT algorithm, as described in Section 3.4.2, is that it requires a very long sensing period ranging from 20 ms up to 100 ms, in order to observe the activity in the medium and decide the LTE “ON” and LTE “OFF” periods. Furthermore, at the end of an LTE “ON” period or at the end of a Wi-Fi opportunity slot during the LTE “ON” period, LTE starts transmitting without sensing the medium for ongoing Wi-Fi transmissions. This results in several collisions among LTE and Wi-Fi. These drawbacks can be eliminated by the use of an energy level pattern periodically transmitted by the eNB. Such a pattern can be sensed by Wi-Fi and other LTE networks in order to achieve inter- and intra-technology synchronization and to adjust their behavior.

In the proposed methodology, we define three different energy level patterns that can be used for different purposes. These patterns are defined as follows:

- Synchronization pattern that enables inter- and intra-technology synchronization and that is transmitted by the first activated network
- LTE identification pattern that is transmitted by the eNB of a newly-activated LTE network in order to inform the rest of the networks about its presence
- Wi-Fi identification pattern that is transmitted by the AP of a newly-activated Wi-Fi network in order to inform the rest of the networks about its presence

**Figure 8:**
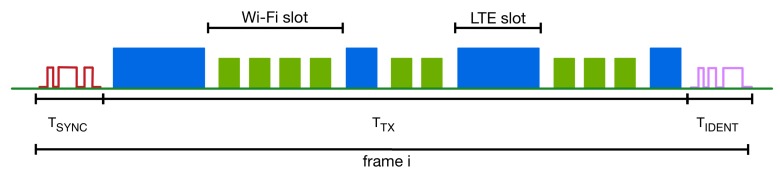
Enhanced CSAT time frame.

Additionally, we define a new time frame as is depicted in Figure 8. This time frame starts with a period T${}_{\mathrm{SYNC}}$ dedicated to transmission and reception of the synchronization pattern. Then, the main part of the frame is called T${}_{\mathrm{TX}}$ and is divided into slots for LTE and Wi-Fi traffic. The last part of the frame is called T${}_{\mathrm{IDENT}}$, and it is used for LTE and Wi-Fi pattern transmission, in order to identify any new networks and adjust the LTE and Wi-Fi slots for the next time frame. Initially, when a new network is activated, it must sense the medium for a period of time equal to a frame in order to discover potential synchronization patterns. If such a pattern does not exist, then the network starts periodically transmitting a synchronization pattern signal to enable inter- and intra-technology synchronization. On the other hand, if the new network senses a synchronization pattern, then it does not initialize a periodic synchronization pattern signal transmission, but it keeps sensing the medium to identify the next synchronization pattern that is expected at the beginning of the next frame. At the moment that two sequentially synchronization patterns are sensed, then the new network can be in synchronization with the rest of the networks. The length of a frame can be stable over time or can vary based on the number of cooperating networks and the amount of transmitted traffic. In the case that a variable frame size is used, then the new network must sense the medium to discover a potential synchronization pattern for a time period that is equal to the maximum frame length.

From the moment that the newly-activated network is in synchronization with the rest of the networks, it must transmit a corresponding LTE or Wi-Fi identification pattern during the next T${}_{\mathrm{IDENT}}$ period. This way, the rest of the networks will be notified for the new LTE or Wi-Fi network. The newly-activated network can identify the LTE and Wi-Fi slots that have already been created by sensing the medium during a T${}_{\mathrm{TX}}$ period. The network that is in charge of transmitting the synchronization pattern transmits its identification pattern only after it senses the first identification pattern from another network during the same T${}_{\mathrm{IDENT}}$ period. If an LTE or Wi-Fi identification pattern is sensed during the T${}_{\mathrm{IDENT}}$, then the LTE and Wi-Fi slots during the T${}_{\mathrm{TX}}$ period are readjusted. This readjustment is done based on the number and the type of the co-located networks. The creation of the new slots will be decided based on the same predefined mechanisms for both LTE and Wi-Fi networks. Moreover, the length of the LTE and Wi-Fi slots will be decided based on the common scheduling mechanism that is used by LTE and Wi-Fi. Furthermore, during a time slot, the eNBs and the APs will measure the channel utilization in order to further adapt the slots for the next frame.

**Figure 9:**
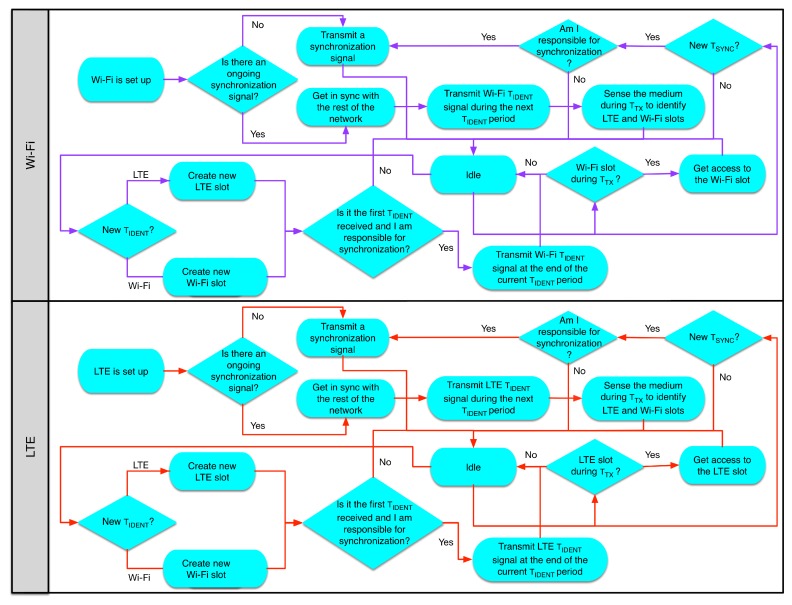
Enhanced CSAT flowchart.

As we mentioned above, the T${}_{\mathrm{TX}}$ phase is divided into Wi-Fi and LTE slots. During the Wi-Fi slots, the different nodes will be able to compete for the medium using the traditional CSMA/CA method, as has been described in Section 3.1.2. On the other hand, during an LTE slot, one or more eNBs can schedule transmissions to their attached UEs. Up to today, much work has been done towards interference mitigation between different eNBs, known as eICIC, which was initially introduced in 3GPP LTE Release 10 [19]. Additionally, eNBs could sense the medium before a transmission to further reduce interference. Figure 9 shows the flowchart of the algorithm described above.

From the description of the method above it is clear that such a cooperation method, for all the advantages that it offers such as intra-technology synchronization, interference management and indirect technology recognition, also has some aspects that require further investigation. For instance, mechanisms should be included that are capable of merging separate co-located networks that have been set up using this cooperation technique or mechanisms that can cope with potentially lost special patterns. This is outside the scope of this paper.

The described technique requires small changes for both LTE eNB and Wi-Fi AP. Firstly, both wireless technologies must be able to transmit, detect and recognize the different energy level patterns that have been described above for synchronization and scheduling purposes. Moreover, the MAC protocols of LTE and Wi-Fi need to be modified in order to create and access the LTE and Wi-Fi slots based on the received T${}_{\mathrm{IDENT}}$.

### 4.3. Enhanced LTE LAA

Cooperation techniques among LTE and Wi-Fi can further enhance the current coexistence techniques such as LTE LAA, providing improved use of the available spectrum towards an enhanced user experience.

3GPP has introduced four different channel access priority classes for DL LTE LAA. Table 2 shows the different priority classes, where the smaller the number of the class, the higher the priority. This table is defined in the 3GPP specifications describing the channel access procedure for LTE LAA [15]. As can be seen from the table, each priority class uses different T${}_{m\cot,p}$, which refers to the maximum channel occupancy time for priority class p. For the priority Classes 3 and 4, T${}_{m\cot,p}$ is 10 ms if the absence of any other co-located technology sharing the same spectrum band can be guaranteed on a long-term basis. In a different case, it is limited to 8 ms. According to the LTE LAA standards, an eNB cannot continuously transmit in the unlicensed spectrum for a period longer than T${}_{m\cot,p}$. On the other hand, when frame aggregation is not used, Wi-Fi transmits only one packet when it gains access to the medium performing CCA. A Wi-Fi packet transmission typically lasts a few hundreds of µs. It is clear that the ratio among LTE and Wi-Fi channel occupancy is not balanced.

**Figure 10:**
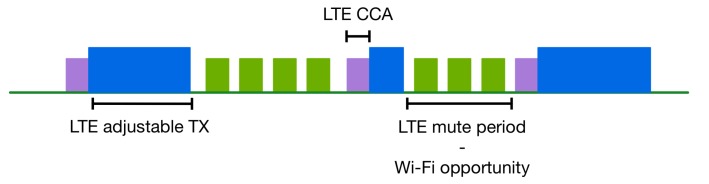
LTE adjustable channel occupancy time and mute period.

To render LTE LAA fairer for Wi-Fi, we propose a scheme according to which LTE will transmit using an adaptable maximum channel occupancy time. Moreover, a variable mute period of LTE-LAA after a transmission can also be introduced, as is depicted in Figure 10, in order to avoid consecutive LTE burst transmissions. The proposed scheme can be further enhanced by introducing cooperation using in-band energy level patterns. In this case, a Wi-Fi or an LTE network can announce its presence by transmitting a corresponding identification special energy pattern. This way, an LTE network will be informed about the presence of other LTE or Wi-Fi networks. LTE can use this information to select and adjust both the burst duration and the mute period.

The decision of the channel occupancy time and the length of the mute period will be made based on a combination of parameters. One important parameter is the number of present Wi-Fi and LTE networks, which have been identified by an identification special energy pattern. This information can assist LTE to keep a balance of channel occupancy in time. Another important parameter that can further enhance this balance is the channel utilization during the silent periods of LTE, meaning the backoff period and the LTE mute period. The LTE must continuously sense the medium to identify the amount of transmitted traffic. Then, it can decide the maximum transmit duration and the length of the mute period. The type of traffic, such as delay-sensitive traffic, can be part of the decision. However, delay-sensitive traffic can be transmitted via the licensed anchor if the unlicensed channel utilization is high. Hence, a quiet channel will result in a short or zero mute period and a high channel occupancy time. On the other hand, an active channel will result in a higher mute period and a shorter occupancy time. It is clear that the algorithm that controls the LTE transmission duration and the LTE mute period is critical. As a first approach, the LTE network can use a slow start type of algorithm. According to such an algorithm, LTE will access the medium for a satisfying portion of time if no other technology is present for a long time. By the time it detects inter-technology coexistence, it will reset back to a minimum value. Then, it will try to find a new balance towards a harmonious coexistence with Wi-Fi.

The method described above requires small modifications to the LAA LTE standards to allow variable mute and channel occupancy periods based on the decisions that the LTE scheduler will make, as well as transmission, reception and interpretation of the special energy patterns.

### 4.4. Advanced Frequency Selection

The aforementioned cooperation techniques can be further enhanced by an advanced frequency selection mechanism. Wi-Fi systems already support DFS (Dynamic Frequency Selection) that can be used by adjacent and non-centrally coordinated APs to avoid interference. DFS is mandatory in the 5250–5350 MHz and 5470–5725 MHz Unlicensed National Information Infrastructure (U-NII) bands of unlicensed spectrum for radar avoidance [31]. Within 3GPP, it has been agreed that a frequency selection functionality is an implementation issue and will not be part of the LTE specifications [32]. An advanced frequency selection mechanism will increase LTE fairness in the unlicensed spectrum and render it compliant with the regional regulations.

In the case of the cooperation technique that is described in Section 4.2, an eNB can sense the activity on different channels in the unlicensed spectrum. If an idle channel is available, it can decide to perform a new frequency selection and either start the transmission of the periodic synchronization pattern, if there is not already one, or it can be synchronized with another existing network that already periodically transmits a synchronization pattern. In order to avoid radio link failure among the eNB and the UEs that are attached to it, the eNB must notify the UEs about the new frequency that will be used, so they can perform the transition synchronously.

The same frequency selection procedure can be adopted regarding the enhanced LTE LAA technique that is described in Section 4.3. An eNB operating in LTE LAA can sense multiple unlicensed channels in order to move to a less busy one. Similar to the first case, the eNB must notify the attached UEs about the new frequency and initiate the transition progress.

### 4.5. Inter-RAT TDMA

**Figure 11:**
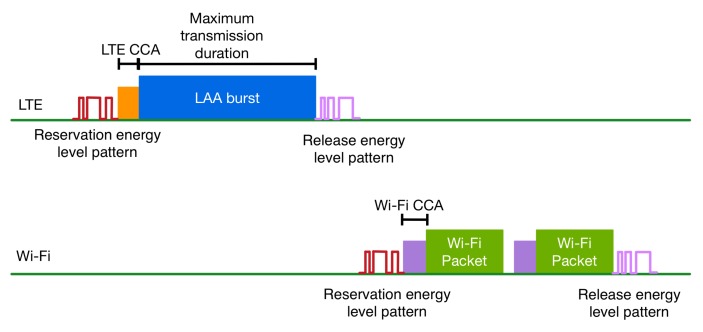
Inter-Radio Access Technology (RAT) TDMA.

The RTS (Request to Send)/CTS (Clear to Send) method has been introduced in the 802.11 standard as an optional feature to control the access to the medium [33]. If RTS/CTS is enabled, then a Wi-Fi node will not transmit until it completes an RTS/CTS handshake. According to this handshake, before a data transmission, a node first transmits an RTS frame to the destination indicating how long the transmission will last. The receiver should reply with a CTS message after an SIFS period. CTS contains a time value that informs other nodes to postpone their transmission during the length of this value. Using the in-band pattern recognition methodology, an inter-RAT RTS/CTS method can be developed among LTE and Wi-Fi, as is depicted in Figure 11. Using such a method, the co-located wireless technologies can reserve the medium for a maximum time duration. This way, they can operate in an inter-technology Time Division Multiple Access (TDMA) way.

In this method, we introduce two different energy level patterns that can be used to reserve and release the medium. These patterns are defined as follows:

- Reservation energy level pattern. This pattern is used by a wireless network to inform other co-located networks that it will reserve the medium for a period of time smaller or equal to a maximum transmission duration.
- Release energy level pattern. This pattern is used by a wireless network to inform other co-located networks that the medium has been released.

Before an LTE or a Wi-Fi transmission, the eNB or the AP tries to gain access to the medium by broadcasting a reservation energy level pattern. When a network detects and interprets such a signal, it postpones its transmissions. The network that transmitted the reservation pattern can gain access to the medium for a period of time smaller or equal to a maximum transmission duration. The choice of the maximum transmission duration that a network can access the medium is very important, as it should guarantee that the medium is not monopolized and every network can access it in a fair way. When the network completes a transmission or when the maximum transmission duration is reached, then it broadcasts a release energy level pattern. At this point, another or the same network can reserve the medium in order to transmit.

During the transmission duration, an eNB schedules resource blocks the UEs that are attached to it, while in case of Wi-Fi, the nodes compete for the medium using the traditional CSMA/CA technique. Before the start of transmission in the reserved timeslot, the network that requests access to it, must sense the medium for ongoing transmissions by networks that are not part of the cooperation mechanism in order to prevent interference with them.

From the above description, it is clear that the implementation of an inter-RAT TDMA mechanism requires small modifications for both LTE and Wi-Fi protocols. These modifications will render both technologies capable of transmitting, detecting and interpreting the in-band energy level patterns, so that they can reserve the medium or postpone their transmissions.

## 5. Indirect Cooperation via a Third-Party Entity

### 5.1. Introduction

The previous section described different cooperation techniques, wherein the participating LTE and Wi-Fi networks can cooperate directly by sending, sensing and interpreting in-band energy patterns. This section goes one step further and discusses the cooperation possibilities, when LTE in the unlicensed spectrum and Wi-Fi are able to exchange messages and express their requirements through a third-party entity. This third-party entity can communicate with both technologies in order to exchange the necessary information that can lead to optimal spectrum usage and enhance the user experience. The third-party entity can be either a central entity, such as a Central Coordination Entity (CCE), or it can be deployed in a distributed manner (Distributed Coordination Entity (DCE)). In the DCE case, a third-party entity must be connected with the base station of each network (LTE eNB or Wi-Fi AP). Thus, the different entities must communicate with each other in order to convey the messages from one network to the other. In this case, the complexity of the cooperation schemes increases. For this reason, in the rest of the article we focus on the usage of the CCE as a third-party entity. However, for the proposed techniques, both a CCE or a DCE can be used.

It is expected that this kind of cooperation can lead to better spectral management results compared to the previously-mentioned techniques in Section 4, as both technologies can explicitly declare their requirements and speak indirectly to each other through the CCE. Such a type of cooperation between LTE and Wi-Fi can offer high flexibility, which can lead to the implementation of advance cooperation techniques. Moreover, cooperation of this type does not increase the complexity of the operation of the LTE and Wi-Fi transmitter and receiver. However, the implementation of the CCE, as well as the requirement for the CCE entity as part of the network adds extra complexity to the design of such a system.

The Wi-Fi APs and the LTE eNBs can be connected to the CCE by either a wired or a wireless link. In the case of a wired connection, the on-demand communication among the networks and the CCE is guaranteed. Of course, wired connectivity limits the flexibility in terms of deployment. These deployment scenarios are limited mainly to indoor deployments, such as in office environments. On the contrary, wireless communication offers higher deployment freedom, but the transmission of the messages between the CCE and the wireless technologies has to deal with the same well-known issues that arise in every wireless communication, such as interference. For simplicity, in the rest of the paper, we assume that there is wired communication among the CCE, the eNBs and the APs.

Furthermore, the CCE must ensure synchronization between the networks that participate in the cooperation scheme. The synchronization among the networks for such cooperation techniques is often critical, as the exchange of information and the access to the medium need to be done in precise time instances. The synchronization requirements depend on each single technique, and therefore, we discuss them in the following subsections.

The rest of this section analytically describes different possible cooperation methodologies that can be applied, when a CCE is available among LTE and Wi-Fi. In addition, for each method, we discuss the changes that are required for both LTE and Wi-Fi protocols.

### 5.2. Adjustment of LTE Transmission Based on Wi-Fi Requirements

As discussed in Section 3.4.1, LTE LAA, in its current form and after a successful CCA, starts transmitting for a duration of 2–10 ms, depending on the channel access priority class to which it belongs (Table 2). Thus, the duration of an LTE transmission is much longer than a typical transmission duration of Wi-Fi (typically a few hundreds of µs), which transmits only one packet after a successful CCA.

In order to balance LTE occupation of the wireless medium versus Wi-Fi, an event-based coordination technique can be applied, which adjusts the LTE transmission duration based on the requirements of Wi-Fi. Additionally, a mute period at the end of an LTE transmission is introduced, as is depicted in Figure 10. Wi-Fi can exploit this period to gain additional access opportunities to the medium resulting in a longer time period of Wi-Fi transmissions.

**Figure 12:**
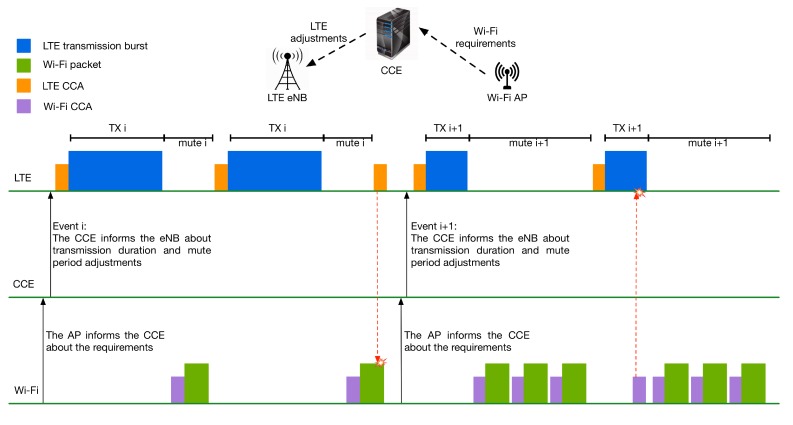
Adjustment of LTE transmission based on Wi-Fi requirements.

The proposed scheme requires that both LTE and Wi-Fi perform a CCA before a transmission. This guarantees that the regional regulations are satisfied and that the cooperation scheme is able to coexist with other potential co-located legacy networks. Figure 12 illustrates the proposed network architecture.

Initially, when a new Wi-Fi network is activated, the AP collects the requirements of the Stations (STAs) that are connected to it and sends them to the CCE. The AP keeps collecting the STAs requirements periodically. Typically, the requirements of a network change on a relatively slow timescale. Thus, this period can be in terms of tens of milliseconds up to a few seconds. When the AP collects new requirements, it checks the variation compared to the previous state reported to the CCE. If this variation exceeds a defined threshold, then the AP reports the new requirements to the CCE. The Wi-Fi AP’s report to the CCE may consist of two different types of information. The first one comprises the summarized statistics of the load of each STA. The second type refers to the future requirements for the next time frame, reported by the STA during the current time frame. These future requirements correspond to a summarized bit rate per second for every Wi-Fi Multimedia (WMM) Access Categories (ACs). For the 802.11 standard, four different WMM priority classes are defined for handling the data traffic regarding the QoS requirements. These classes are the following:

- AC_BK (background traffic)
- AC_BE (best effort traffic)
- AC_VI (video traffic)
- AC_VO (voice traffic)

After the CCE receives the new requirements, it evaluates them, and if a change of LTE transmission behavior is needed, it triggers an event and informs LTE about the new transmission duration and the new length of the mute period. Hence, the next LTE transmission will be done according to the new configuration. In the case of multiple LTE networks, the transmission duration and mute period should be synchronized among them. This way, a scenario according to which an LTE transmission occurs during the mute period of another LTE network occupying the Wi-Fi grant can be avoided.

This way, under a heavily loaded Wi-Fi network or if Wi-Fi needs to transmit delay-sensitive traffic, the coordinator schedules a shorter transmission duration for LTE and/or a longer mute period after a transmission. The ratio of LTE transmission duration and LTE mute duration must be chosen carefully, so that LTE does not suffer from continuous short transmission durations and long mute timeslots in the unlicensed spectrum. Hence, CCE must keep a record of the last configurations of the LTE transmission and mute duration over time. This way, it will be able to maintain a balance between serving the Wi-Fi requirements and giving equal channel access opportunities to all of the cooperating networks.

For the cooperation technique that is described in this section, no synchronization is needed between Wi-Fi and LTE, since there is no need for synchronized access to the medium between the different technologies.

The implementation of such a cooperation scheme requires some changes to both LTE and Wi-Fi. Regarding LTE, it needs to be extended in a way that it can receive and use the transmitted messages by the CCE. This extension also includes the introduction of the additional mute period after a transmission in the unlicensed spectrum and variable transmission period. On the other hand, the Wi-Fi STA part has to be extended to be capable of reporting the transmission requirements to the AP. Additionally, the AP must be able to collect, evaluate and report these requirements to the CCE. Finally, a sophisticated CCE needs to be employed. This CCE must be able to collect the requirements from the Wi-Fi AP, process them and inform the eNB about the new configuration parameters. The described cooperation scheme targets serving the Wi-Fi requirements by adjusting the transmission duration of LTE, concurrently maintaining equal channel access opportunities among the participating networks. This technique can be implemented with a relatively small effort, as it requires small modifications for LTE and Wi-Fi as described above. On the other hand, it does not take into account the requirements of LTE. In the next section, a more complex, but enhanced technique is proposed, in which the CCE schedules LTE and Wi-Fi transmissions, considering the requirements of both networks.

### 5.3. Adjustment of LTE and Wi-Fi Transmission Based on Requirements and History

In a more sophisticated approach than the technique described in the previous section, the CCE adjusts both LTE and Wi-Fi transmission timeslots based on their requirements, as well as the channel activity history.

In order to control the duration and the frequency of LTE bursts, we assume variable transmission duration, followed by a variable mute period, in an event-based way similarly to the previous technique. Furthermore, both LTE and Wi-Fi networks must perform a CCA before a transmission in order to be compliant with the regional regulations and respect potential transmissions from other networks that do not participate in the cooperation scheme.

In a similar way to the previous section, when a new Wi-Fi or LTE network is activated, the AP or the eNB collects and sends the network requirements to the CCE. Then, the AP or the eNB keeps collecting requirements periodically. This period can be in terms of tens of milliseconds up to a few seconds and can vary between different networks. The new requirements are compared to the previous state reported to the CCE. If the variation exceeds a defined threshold, then they are sent to the CCE. The Wi-Fi requirements are the ones that have been described in Section 5.2. Similarly, the LTE eNB informs the CCE about the transmission load of the LTE network.

When the coordinator receives the requirements, then it decides about the LTE transmission duration and mute period based on the current requirements (e.g., summarized bit rate per second) and the history of transmissions. The history of transmissions represents a moving time window that tracks the average channel utilization for each participating network. The decision can be made, taking into account different weights of the history and the current requirements. To this end, the values of *p* and 1−*p*, where 0≤p≤1, are used to express the weights of the current requirements and history that will be used respectively. Initially, history records are not available. Hence, only the current requirements are taken into consideration.

**Figure 13:**
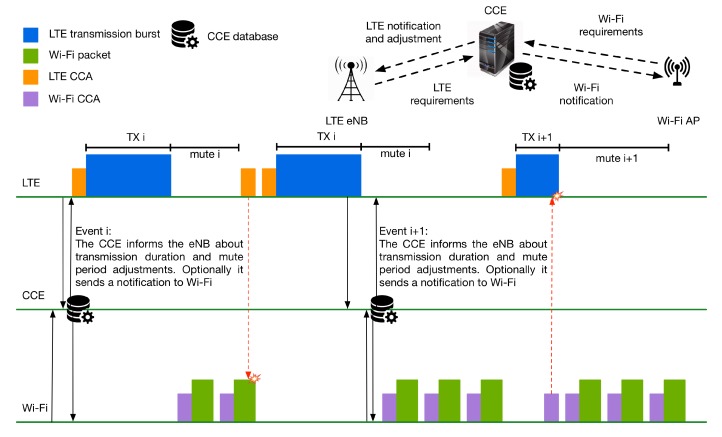
Adjustment of LTE and Wi-Fi transmission based on requirements and history.

The weights of the current requirements and history will be used by the scheduler of CCE to compute the new duration of the LTE transmission and the new duration of the LTE mute period. Hence, when a network must transmit mainly delay-sensitive traffic, then the weight of the current requirements will be higher than the weight of history, as in this case, the traffic must be delivered on time. In a similar way, when best-effort traffic has to be transmitted, then the history of the channel utilization may have a higher weight in the final decision. If a change to the transmission behavior of LTE is needed, then the CCE triggers an event and informs LTE about the new configuration of transmission. The next LTE transmission will be done according to the new configuration. Again, in case of multiple eNBs, the transmission duration and mute period should be synchronized. Figure 13 presents the aforementioned cooperation technique.

**Figure 14:**
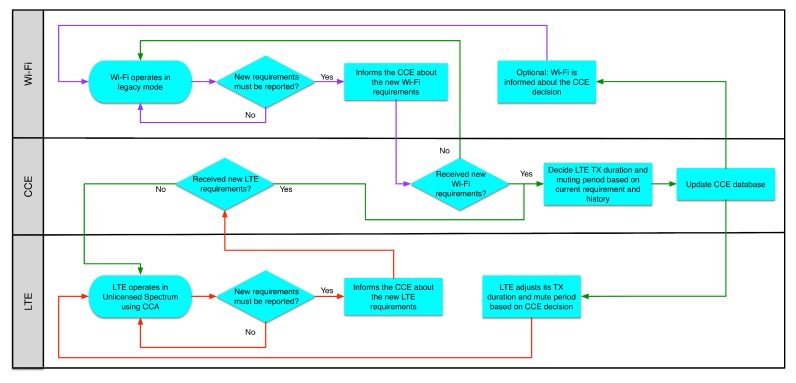
Flowchart of LTE and Wi-Fi transmissions adjustment by Central Coordination Entity (CCE).

The flowchart of the proposed cooperation method is shown in Figure 14. When needed, the eNB and the AP inform the coordinator about their new requirements. When the coordinator receives the requirements, it evaluates them and decides if modifications to the LTE transmission duration and mute period are required, as described above. Then, the coordinator informs the participating networks about its decision and updates its database. Based on the coordinator’s decision, the LTE adjusts its transmission duration and the mute duration for the next time frame giving the necessary slots to Wi-Fi.

In this technique, synchronization between Wi-Fi and LTE is not critical, as there is no need for synchronized access to the medium between the wireless technologies that participate in the cooperation scheme.

Similar to the method described in Section 5.2, such a cooperation mechanism requires changes for both LTE and Wi-Fi, so they can inform the CCE about their requirements. Additionally, extensions are required to eNB and AP, so they can receive and use the information transmitted by the CCE. Finally, the CCE itself has extra complexity compared to the previous method, as it needs to keep track of the transmission for all of the participating networks and it has to decide about the sharing of the channel based on complex criteria.

### 5.4. Negotiation between LTE and Wi-Fi

**Figure 15:**
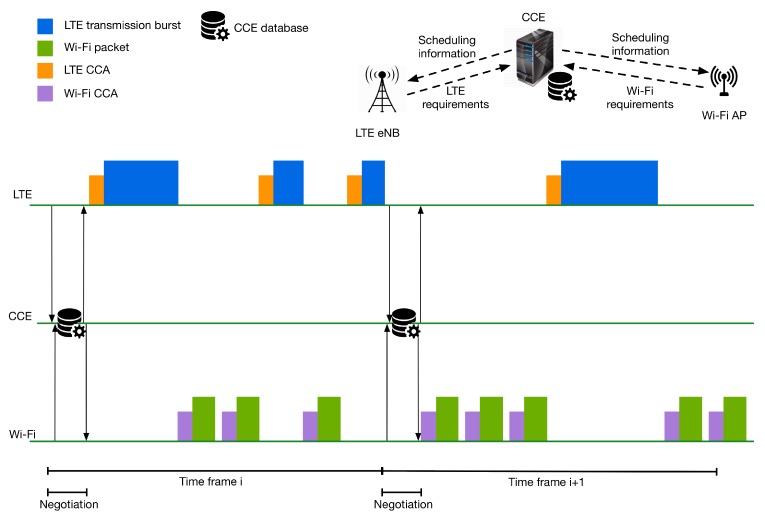
Negotiation phase between LTE and Wi-Fi and timeslot assignment.

The method described in the previous section gives to the coordinator the possibility to decide, in the beginning of a time frame, the duration of the LTE transmissions and the following mute period. These durations remain stable during the whole time frame duration. In a different approach, the CCE can schedule variable durations of LTE transmission and mute periods in the same time frame. This technique can offer higher transmission flexibility and can better serve the QoS requirements as the networks can access the medium in a more dynamic way. The proposed methodology is illustrated in Figure 15.

As can be seen, the time domain is divided into time frames with a range from tens of milliseconds up to a few seconds. In the beginning of each frame, there is a negotiation phase during which the participant networks of the cooperation scheme report their requirements to the coordinator. As presented in Section 5.3, the coordinator keeps a record of the channel activity. Hence, it knows the percentage that each network has occupied the channel in the past. When the coordinator receives the requirements during the negotiation phase, it divides the time frame into slots and assigns them to the different networks based on their demands and the history of the channel activity. The proportion of the current requirements and history will be decided in an advanced way, similar to the one described in Section 5.3. In this technique, the wireless technologies access the medium in an inter-technology TDMA way. When a timeslot is assigned to an LTE network, the eNB schedules resources for the UEs that are attached to it. On the other side, if a timeslot is assigned to a Wi-Fi network, then the Wi-Fi nodes are competing for the medium using the traditional CSMA/CA method. Before a transmission, both LTE and Wi-Fi networks must perform a channel estimation to ensure that the channel is free from potential ongoing transmissions by other networks that do not participate in the cooperation mechanism. If the channel is sensed as busy, then the network continues to sense the wireless medium till the end of the assigned slot. If the medium becomes idle, the corresponding network starts a transmission for the remaining time. The CCE can take into account such potential cases for future scheduling decisions in order to assign longer or more slots to the network that missed one or more assignments during the previous frame(s).

In this technique, the coordinator needs to ensure synchronization of the participating networks in a frame time domain. This way, the networks will be able to express their requirements and negotiate about the spectral requirements during the negotiation phase. Additionally, the participating networks will be able to access the corresponding assigned LTE and Wi-Fi time slots that the scheduler creates in a synchronized manner.

Such a cooperation mechanism requires modifications of LTE and Wi-Fi, so they can express their requirements during the negotiation phase. Moreover, the networks should be able to interpret the messages sent by the CCE and transmit only during the timeslots that have been assigned to them by the coordinator. Additionally, a sophisticated CCE needs to be implemented, which will be able to receive the requirements from different networks and to assign timeslots using advanced methods, taking into account the current requirements and the history of the channels’ occupancy.

## 6. Comparison of the Proposed Schemes

This section highlights the main differences between the proposed techniques that have been discussed in the previous sections. Table 3 summarizes these differences in terms of the modifications that each technique requires, the synchronization requirements, the complexity and the performance of each cooperation scheme.

The complexity of each technique is divided into two different aspects named expected implementation complexity and information exchange overhead. The expected implementation complexity indicates the number of modifications to the current standards and protocols of each wireless technology that each proposed technique requires. For instance, the “Enhanced LTE LAA” technique requires only a few changes to the LTE LAA standards in order to allow variable mute and transmission opportunity periods, as well as transmission, reception and interpretation of the special energy patterns. On the other hand, the “adjustment of LTE and Wi-Fi transmission based on requirements and history” technique requires an average implementation effort, as it involves a sophisticated coordinator unit that must be capable to communicate with the co-located networks and tune the LTE parameters based on different parameters (current requirements and history).

The information exchange overhead expresses the amount of information or signals that are exchanged between the co-located wireless networks towards the attainment of cooperation according to each technique. For example, the “Inter-RAT TDMA” cooperation scheme requires the exchange of several energy level patterns between the co-located networks, so that they can reserve and release the medium prior to and after a transmission. In contrast, according to the “adjustment of LTE transmission based on Wi-Fi requirements” technique, a Wi-Fi network informs the coordinator about its requirements in an event-based manner and in a relatively slow timescale (tens of milliseconds up to a few seconds). Thus, such technique is expected to have a low information exchange overhead.

Furthermore, the performance of each proposed cooperation scheme is divided into two aspects named degree of cooperation and expected spectral efficiency. The degree of cooperation indicates the eagerness of each technique to cooperate. The lower the degree of cooperation, the lower the performance of the cooperation technique. For instance, the “negotiation between LTE and Wi-Fi” technique offers a high degree of cooperation, as the participating networks exchange information and negotiate about the spectral requirements towards the best possible serving of their QoS requirements. In contrast, the ”inter-RAT TDMA” technique offers a low degree of cooperation as a network simply reserves the medium for the shortest possible period in an altruistic manner.

The expected spectral efficiency indicates the expected capability of each proposed technique to manage and share the spectrum between the different co-located wireless technologies in an efficient way. For instance, the “negotiation between LTE and Wi-Fi” technique is expected to have a high spectral efficiency, as it selects variable LTE and Wi-Fi slots based on the negotiation result between the co-located technologies.

Similar to the standalone operation of wireless technology, where there is no other competitor for the wireless resources, the number of the nodes operating in each technology will have an impact on the spectral efficiency that each technique can provide. Regarding the LTE network, if there are many UEs, then the resources will be divided in to the different UEs by the LTE scheduler. When there are multiple Wi-Fi nodes, then more nodes would compete during the LTE idle slots decreasing the provided spectral efficiency. In this case, a TDMA channel access scheme for Wi-Fi could improve the achieved spectral efficiency. Additionally, an increased number of nodes corresponds to a higher amount of information or signals that have to be exchanged between the networks (or between the networks and the third-party entity) according to each cooperation technique. Hence, the higher the number of the nodes operating in each technology, the higher the information exchange overhead and the complexity of the cooperation schemes.

## 7. Conclusions and Future Work

Towards 5G, the number of HetNets is expected to increase rapidly. These HetNets consist of different well-established wireless technologies that operate next to each other. Each of these technologies has its own user target group, as it is suitable for specific applications (sensor networks, D2D communications, M2M communications, etc.). Among them, Wi-Fi is the most popular and widely-used wireless technology in the unlicensed spectrum. Recently, LTE in the unlicensed spectrum has been introduced, as it is a technology that can play an important role in dealing with the tremendous wireless traffic increment. Hence, scenarios in which co-located LTE and Wi-Fi networks operate in the same band will soon become very common. Based on this fact, the research community needs to look into cooperation techniques among different technologies in order to use the wireless spectrum as efficiently as possible.

In this article, we describe different cooperation techniques that can be applied between co-located LTE and Wi-Fi networks. These techniques are classified into two main categories. According to the first one, the networks cooperate directly by sending, receiving and interpreting in-band special patterns. In the second category, the cooperating networks can intercommunicate indirectly using a third-party entity such as a CCE. Each technique requires different implementation effort and offers different cooperation flexibility and spectral efficiency. Subsequently, for each proposed technique we analyze the open issues and challenges, as well as the required changes to the LTE and Wi-Fi protocols taking into account regional regulations.

The concepts that are described in this article will be used as a cornerstone for our future work. In the near future, this work can be extended towards the implementation and the comparison of the proposed techniques by initially performing simulations according to the performance indicators as they are mentioned in Table 3. Further, implementation and evaluation based on real hardware can also be done using the LTE and Wi-Fi infrastructure of the W-iLab2 testbed at imec. This way, each cooperation technique can be examined in detail and the analytical results of the provided fairness and spectral efficiency can be obtained. This work can further contribute to the ongoing research and standardization towards an efficient and fair spectral sharing between LTE and Wi-Fi.

## Figures and Tables

**Table 1:** Regional requirements for the unlicensed spectrum.

Frequency/Region		2.4 GHz	5150–5250 MHz	5250–5350 MHz	5470–5725 MHz	5725–5850 MHz
EU	Coexistence	Listen Before Talk, Maximum Transmit (TX) power, Emission mask				
	Protect incumbent	-	Indoor	Indoor/Outdoor	Indoor/Outdoor	-
				DFS/TPC	DFS/TPC	
USA	Coexistence	FCC Part 15.247, 15.401-407, Maximum TX power, Emission mask				
	Protect incumbent	-	Indoor	Indoor/Outdoor	Indoor/Outdoor	Indoor/Outdoor
				DFS/TPC	DFS/TPC	
China	Coexistence	Maximum TX power, Emission mask				
	Protect incumbent	-	Indoor	Indoor DFS/TPC	-	Indoor/Outdoor
Japan	Coexistence	Listen Before Talk, Maximum burst length (4 ms), Maximum TX power,				
		Maximum antenna gain, Emission mask				
	Protect incumbent	-	Indoor	Indoor DFS/TPC	Indoor/Outdoor	-
					DFS/TPC	
Korea	Coexistence	Maximum TX power, Maximum antenna gain, Emission mask				
	Protect incumbent	-	Indoor	Indoor/Outdoor	Indoor/Outdoor	Indoor/Outdoor
				DFS/TPC	DFS/TPC (5470–5650)	

**Table 2:** LTE LAA channel access priority classes.

Channel Access Priority Class (p)	m${}_{p}$	CW${}_{\min,p}$	CW${}_{\max,p}$	T${}_{m\cot,p}$	Allowed CW${}_{p}$ sizes
1	1	3	7	2 ms	3, 7
2	1	7	15	3 ms	7, 15
3	3	15	63	8 or 10 ms	15, 31, 63
4	7	15	1023	8 or 10 ms	15, 31, 63, 127, 255, 511, 1023

**Table 3:** Comparison of the different proposed cooperation schemes.

Cooperation Type	Cooperation Technique	Modifications Required	Sync Required	Complexity		Performance	
				Expected Implementation Complexity	Information Exchange Overhead	Degree of Cooperation	Expected Spectral Efficiency
Direct cooperation via in-band energy level patterns	Enhanced CSAT	Transmission, detection and recognition of special energy level patterns for LTE and Wi-Fi	Between the different networks	Low	Medium	Low	Low
		Variable mute and transmission period for LTE					
		MAC layer modifications for LTE and Wi-Fi					
	Enhanced LTE LAA	Transmission, detection and recognition of special energy level patterns for LTE and Wi-Fi	No	Low	Medium	Low	Low
		Variable mute and transmission period for LTE					
		MAC layer modifications for LTE and Wi-Fi					
	Advanced frequency selection	Modifications similar to the previous two methods	No	Medium	Medium	Low	Medium
		Frequency selection procedures for LTE					
	Inter-RAT TDMA	Transmission, detection and recognition of special energy level patterns for LTE and Wi-Fi	No	Low	High	Low	High
		Variable mute and transmission period for LTE					
		MAC layer modifications for LTE and Wi-Fi					
Indirect cooperation via a third-party entity	Adjustment of LTE transmission based on Wi-Fi requirements	CCE procedures	Between multiple eNBs	Medium	Low	Medium	Medium
		Communication between CCE, LTE eNB and Wi-Fi AP					
		Variable mute and transmission period for LTE					
		MAC layer modifications for LTE and Wi-Fi					
	Adjustment of LTE and Wi-Fi transmission based on requirements and history	CCE procedures	Between multiple eNBs	Medium	Low	High	High
		Communication between CCE, LTE eNB and Wi-Fi AP					
		Variable mute and transmission period for LTE					
		MAC layer modifications for LTE and Wi-Fi					
	Negotiation between LTE and Wi-Fi	CCE procedures	Between the different networks	Medium	Low	High	High
		Communication between CCE, LTE eNB and Wi-Fi AP					
		Variable mute and transmission period for LTE					
		MAC layer modifications for LTE and Wi-Fi					

## References

[1] Qualcomm (2012). Rising to Meet the 1000x Mobile Data Challenge.

[2] Cisco (2015). Cisco Visual Networking Index: Forecast and Methodology, 2014–2019.

[3] 3GPP News (2015). Evolution of LTE in Release 13. http://www.3gpp.org/news-events/3gpp-news/1628-rel13.

[4] 3GPP TR 36.808 (2012). Evolved Universal Terrestrial Radio Access (E-UTRA); Carrier Aggregation; Base Station (BS) Radio Transmission and Reception.

[5] MulteFire Alliance. http://www.multefire.org/.

[6] Huawei White Paper (2014). U-LTE: Unlicensed Spectrum Utilization of LTE.

[7] Maglogiannis V., Naudts D., Willemen P., Moerman I. Impact of LTE Operating in Unlicensed Spectrum on Wi-Fi Using Real Equipment. Proceedings of the IEEE Global Telecommunications Conference (GLOBECOM) 2016.

[8] 3GPP TS 36.211 (2015). Evolved Universal Terrestrial Radio Access (EUTRA); Physical Channels and Modulation;.

[9] IEEE Standard 802.11 (2012). Wireless LAN Medium Access Control (MAC) and Physical Layer (PHY) Specifications.

[10] ETSI EN 301 893 v1.7.1 (2012). Broadband Radio Access Networks (BRAN); 5 GHz High Performance RLAN; Harmonized EN Covering the Essential Requirements of Article 3.2 of the R&TTE Directive.

[11] European FP7 FLEX Project Portal. http://www.flex-project.eu.

[12] imec. https://www.imec-int.com/en/home.

[13] Ratasuk R., Uusitalo M.A., Mangalvedhe N., Sorri A., Iraji S., Wijting C., Ghosh A. License-exempt LTE deployment in heterogeneous network. Proceedings of the IEEE International Symposium on Wireless Communication Systems (ISWCS) 2012.

[14] Nihtilä T., Tykhomyrov V., Alanen O., Uusitalo M.A., Sorri A., Moisio M., Iraji S., Ratasuk R., Mangalvedhe N. System performance of LTE and IEEE 802.11 coexisting on a shared frequency band. Proceedings of the IEEE Wireless Communications and Networking Conference (WCNC) 2013.

[15] 3GPP TS 36.213 V13.3.0 (2016). Evolved Universal Terrestrial Radio Access (E-UTRA); Physical Layer Procedures.

[16] Kwon H.J., Jeon J., Bhorkar A., Ye Q., Harada H., Jiang Y., Liu L., Nagata S., Ng B.L., Novlan T. (2016). Licensed-Assisted Access to Unlicensed Spectrum in LTE Release 13. IEEE Commun. Mag..

[17] Qualcomm (2015). Making the Best Use of Unlicensed Spectrum for 1000x.

[18] Al-Khansa R., Artail H. A Semi-Distributed LTE-Wi-Fi System Design for Future LTE-Unlicensed Deployments in Small-cell Environments. Proceedings of the IEEE Wireless and Mobile Computing, Networking and Communications (WiMob) 2015.

[19] 3GPP TS 36.300 (2010). Evolved Universal Terrestrial Radio Access (E-UTRA) and Evolved Universal Terrestrial Radio Access Network (E-UTRAN); Overall Description; Stage 2.

[20] Zhou Z., Teng F., Liu J., Xiao W. Performance Evaluation for Coexistence of LTE and WiFi. Proceedings of the Computing, Networking and Communications (ICNC) 2016.

[21] Sagari S., Seskar I., Raychaudhuri D. Modelling the Coexistence of LTE and WiFi Heterogeneous Networks in Dense Deployment Scenarios. Proceedings of the 2015 IEEE International Conference on Communication Workshop (ICCW).

[22] Rupasinghe N., Güvenç İ. Reinforcement Learning for Licensed-Assisted Access of LTE in the Unlicensed Spectrum. Proceedings of the Wireless Communications and Networking Conference (WCNC) 2015.

[23] Galanopoulos A., Foukalas F., Tsiftsis T.A. (2016). Efficient Coexistence of LTE with WiFi in the Licensed and Unlicensed Spectrum Aggregation. IEEE Trans. Cogn. Commun. Netw..

[24] Kim C.K., Yang C.S., Kang C.G. Adaptive Listen-Before-Talk (LBT) Scheme for LTE and Wi-Fi Systems Coexisting in Unlicensed Band. Proceedings of the 13th IEEE Annual Consumer Communications and Networking Conference (CCNC) 2016.

[25] Mushunuri V., Panigrahi B., Rath H.K., Simha A. Fair and Efficient Listen Before Talk (LBT) Technique for LTE Licensed Assisted Access (LAA) Networks. Proceedings of the IEEE 31st International Conference on Advanced Information networking and Applications (AINA) 2017.

[26] Hao F., Yongyu C., Li H., Zhang J., Quan W. Contention Window Size Adaptation Algorithm for LAA-LTE in Unlicensed Band. Proceedings of the International Symposium on Wireless Communications Systems.

[27] Hasan C., Marina M.K., Challita U. On LTE-WiFi Coexistence and Inter-Operator Spectrum Sharing in Unlicensed Bands: Altruism, Cooperation and Fairness. Proceedings of the 17th ACM International Symposium on Mobile Ad Hoc Networking and Computing.

[28] Sagari S., Baysting S., Saha D., Seskar I., Trappe W., Raychaudhuri D. Coordinated dynamic spectrum management of LTE-U and Wi-Fi Networks. Proceedings of the IEEE International Symposium on Dynamic Spectrum Access Networks.

[29] Al-Dulaimi A., Al-Rubaye S., Ni Q., Sousa E. (2015). 5G Communications Race: Pursuit of More Capacity Triggers LTE in Unlicensed Band. IEEE Veh. Technol. Mag..

[30] Chen B., Chen J., Gao Y., Zhang J. (2017). Coexistence of LTE-LAA and Wi-Fi on 5 GHz With Corresponding Deployment Scenarios: A Survey. IEEE Commun. Surv. Tutor..

[31] (2003). 5 GHz Agreement. https://www.ntia.doc.gov/legacy/ntiahome/press/2003/5gHzAgreement.htm.

[32] 3GPP TR 36.889 v13.0.0 (2015). Feasibility Study on Licensed-Assisted Access to Unlicensed Spectrum.

[33] IEEE Standard (1999). Wireless LAN Medium Access Control (MAC) and Physical Layer (PHY) Specifications.

